# Solution-Induced Degradation of the Silicon Nanobelt Field-Effect Transistor Biosensors

**DOI:** 10.3390/bios14020065

**Published:** 2024-01-25

**Authors:** Jung-Chih Lin, Zhao-Yu Zhou, Yi-Ching Cheng, I-Nan Chang, Chu-En Lin, Chi-Chang Wu

**Affiliations:** 1 Department of Integrated Chinese and Western Medicine, Chung Shan Medical University Hospital, and School of Medicine, Chung Shan Medical University, Taichung 40201, Taiwan; cshy1027@csh.org.tw; 2 Department of Electronic Engineering, National Chin-Yi University of Technology, Taichung 411030, Taiwan; s4b113001@student.ncut.edu.tw (Z.-Y.Z.); a3242@ncut.edu.tw (Y.-C.C.); 3 Department of Electronic Engineering, Feng Chia University, Taichung 40724, Taiwan; enchung@fcu.edu.tw

**Keywords:** silicon nanobelt, NBFET, nanobelt biosensor, degradation, ion penetration, surface functionalization

## Abstract

Field-effect transistor (FET)-based biosensors are powerful analytical tools for detecting trace-specific biomolecules in diverse sample matrices, especially in the realms of pandemics and infectious diseases. The primary concern in applying these biosensors is their stability, a factor directly impacting the accuracy and reliability of sensing over extended durations. The risk of biosensor degradation is substantial, potentially jeopardizing the sensitivity and selectivity and leading to inaccurate readings, including the possibility of false positives or negatives. This paper delves into the documented degradation of silicon nanobelt FET (NBFET) biosensors induced by buffer solutions. The results highlight a positive correlation between immersion time and the threshold voltage of NBFET devices. Secondary ion mass spectrometry analysis demonstrates a gradual increase in sodium and potassium ion concentrations within the silicon as immersion days progress. This outcome is ascribed to the nanobelt’s exposure to the buffer solution during the biosensing period, enabling ion penetration from the buffer into the silicon. This study emphasizes the critical need to address buffer-solution-induced degradation to ensure the long-term stability and performance of FET-based biosensors in practical applications.

## 1. Introduction

Biosensors are analytical devices that integrate biological recognition elements with transducers to detect and quantify specific analytes in various sample matrices [1,2,3]. These devices are widely used in a range of applications, including disease detection, medical diagnostics, drug selection, and environmental monitoring [4,5,6,7]. Recently, biosensors based on semiconductor materials have attracted more and more attention. These kinds of biosensors offer numerous advantages over traditional analytical techniques, including high sensitivity, high specificity, and miniaturing capability [8,9].

Numerous two-dimensional (2D) semiconductor materials, such as nanowires, nanobelts, and nanotubes, find application in biosensing [10,11,12,13,14]. These 2D materials are used in devices serving as the transmission channel for carriers. Transducer devices employing these materials have demonstrated exceptional properties for detecting trace biomolecules, showcasing ultrahigh sensitivity. However, the critical challenge lies in addressing the stability and potential degradation induced by the solution when operating, given that these materials are exposed to ionic solutions [15,16]. Ensuring the accurate and reliable performance of biosensors over extended periods becomes imperative, despite their ultrahigh sensitivity. The solution-induced stability and degradation of biosensors made from 2D materials necessitate careful consideration, emphasizing the need to develop strategies that safeguard their performance in the presence of ionic solutions.

Biomedical sensors are designed to detect trace biomolecules within solutions, which can comprise various body fluids like blood, serum, urine, or buffered solutions [17,18]. These biosensors are subject to degradation over time due to various factors, including environmental conditions, surface chemistry, and biological interactions. Surface chemistry is one of the critical factors that can affect the stability of biosensors. The surface chemistry of the transducer material can significantly influence the interaction between the biological recognition element and the analyte [19]. The adsorption of proteins, nucleic acids, and other biomolecules onto the transducer surface can interfere with the sensing mechanism and cause false reading results.

The stability issue stands as a pivotal concern when considering the performance of a transistor device. This concern becomes particularly pronounced when dealing with biosensor devices, as they are frequently employed in liquid environments. Unlike conventional transistor devices that typically operate in controlled environments safeguarded against moisture, light, and magnetic interference, biosensors must function within the context of trace biomolecule detection in solutions. These solutions often comprise various body fluids, such as blood, serum, urine, or buffer solutions. The extended immersion of a biosensor in such solutions can accelerate the degradation of the transistor device itself, or even lead to the deterioration of its surface functionalization [20,21]. What further compounds the challenge is the high ion concentration present in these body fluids or buffer solutions. When a semiconductor biosensor device is immersed in such solutions, these ions can permeate the device, raising concerns related to the device’s reliability [22,23].

In light of these considerations, it becomes imperative to conduct a time-dependent stability assessment of the 2D material-based biosensors when immersed in a buffer solution. Such an evaluation is vital to ascertain the device’s ability to maintain consistent and reliable performance over time. The literature concerning solution-induced stability is, however, scarce. Therefore, this study aims to uncover the longevity and resilience of the biosensor device when subjected to the complex and dynamic conditions of a liquid environment.

This paper details the fabrication of a silicon nanobelt field-effect transistor (NBFET) device using a commercially available CMOS-compatible manufacturing technique. Diverging from traditional planar FET devices, the NBFET device employs a narrow and thin nanobelt as a channel to establish the connection between the source and drain. This unique design imparts ultrahigh sensitivity to the NBFET biosensor, particularly in the detection of biomolecules. The fabricated NBFET device was specifically employed for the detection of DNA strands. Subsequently, this NBFET biosensor underwent immersion in a buffer solution, and its electrical properties were systematically monitored to assess the solution-induced stability of the device. This investigation into the device’s performance over prolonged periods in challenging environments provides valuable insights into its durability and efficacy as a stable and robust sensing tool. The outcomes of this study not only contribute to the broader field of biosensors but also establish a foundational reference for future advancements in biomedical devices. By elucidating the solution-induced stability of the NBFET biosensor, this research paves the way for improved understanding and innovation in the development of reliable and enduring sensing technologies for various biomedical applications.

## 2. Materials and Methods

A side-gated NBFET was fabricated and applied as a biosensor device. The sensor was completed using a commercially available CMOS process. The whole sensing procedure was conducted in the Taiwan Semiconductor Research Institute (TSRI). The chemicals, reagents, and solvents used in this study were reagent-grade quality or higher. The experimental procedure was divided into five parts: (1) chemicals and materials; (2) fabrication of the side-gated NBFET device; (3) surface functionalization and biografting; (4) fabrication of the microfluidic channel; (5) device measurement and analysis. These five parts are discussed in more detail in the following section.

### 2.1. Chemicals and Materials

Analytical-grade ethanol (C_2_H_5_OH, ≥99.8% in purity), 3-amino-propyl-triethoxy-silane (APTES; H_2_N(CH_2_)_3_Si(OC_2_H_5_)_3_, 221.37 g/mol), glutaraldehyde (GA; OHC(CH_2_)_3_CHO; 100.12 g/mol), and phosphate-buffered saline (PBS, 120 mM NaCl, 2.7 mM KCl, and 10 mM phosphate buffer; pH 7.4) were purchased from Sigma-Aldrich (St. Louis, MO, USA). A synthetic fluorescein isothiocyanate (FITC)-labeled DNA strand having the sequence of 5′-NH_2_-ACGTCCCGCGCAGGA-3′-FITC was purchased from Blossom Biotechnologies, Taipei, Taiwan. To prevent chemical deterioration, APTES was stored in a 4 °C environment, and DNA and GA were stored in a −20 °C environment after dilution.

### 2.2. Fabrication of the Silicon NBFET

**Figure 1:**
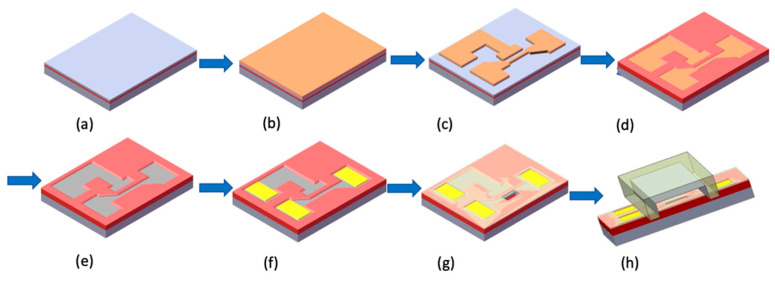
Schematic diagram of the fabricating flowchart of the NBFET device. (**a**) SOI wafer as the substrate. (**b**) SiO_2_/Si_3_N_4_ stack film deposition. (**c**) Active region definition. (**d**) Thick SiO_2_ film deposition. (**e**) SiO_2_/Si_3_N_4_ stack film removal. (**f**) AlSiCu electrode pad deposition and definition. (**g**) Si_3_N_4_/TEOS-oxide film deposition and etching-back to expose the nanobelt. (**h**) Microfluidic channel installation for bio-sensing.

A schematic diagram of the fabricating flowchart of the NBFET device is depicted in Figure 1. To form the silicon NBFET sensors, commercially available 6 in. (100) silicon-on-insulator (SOI) wafers were used as the substrate (Figure 1a). Firstly, a stack of films of 30 nm thick TEOS-oxide (SiO_2_) and 100 nm thick silicon nitride (Si_3_N_4_) was deposited sequentially on the substrate (Figure 1b), followed by the 1st lithography and etch process to define the stacked films as an active region of the device (Figure 1c). The lithography system used in this experiment was a TRACK (TEL CLEAN-TRACK MK8, Tokyo Electron Ltd., Tokyo, Japan) and I-line exposure system (Canon FPA-3000i5 stepper, Canon Inc., Tokyo, Japan). The wafers were thermally grown for oxidation, transforming the exposed silicon substrate into a 500 nm thick SiO_2_ film (Figure 1d). On the other hand, the SiO_2_/Si_3_N_4_ film stack prevented the underlying silicon from oxidation, and therefore the silicon film remained. This result was caused by the nature of the high-density Si_3_N_4_ film. This local oxidation of silicon is often used as the isolation technique in MOS manufacturing. In addition, the linewidth of nanobelts shrunk due to the oxidant diffusing laterally during this process. This phenomenon could induce the linewidth of nanobelts to be smaller than the critical width of the exposure system’s capability.

The SiO_2_/Si_3_N_4_ stack film was removed after the oxidation process, and therefore a nanobelt was formed (Figure 1e). The 2nd lithography process was then conducted, followed by As+ ion implantation and rapid thermal annealing to form the source, drain, and gate region. A stacked layer of 100 nm thick TEOS-oxide and 20 nm thick Si_3_N_4_ was deposited sequentially to protect the device, and then the 3rd lithography and etch process was used to form a contact hole. A 200 nm thick AlSiCu alloy film was deposited using a sputtering system, followed by the 4th lithography and metal etch process to define the electrode pad (Figure 1f). Finally, the 5th lithography and Si_3_N_4_/TEOS-oxide film etch-back process was conducted to expose the nanobelts for biosensing purposes (Figure 1g). To avoid the nanobelts degenerating, the completed biosensor devices were coated with a photo-resist and stored in a clean room before use.

### 2.3. Surface Functionalization and Biografting

**Figure 2:**
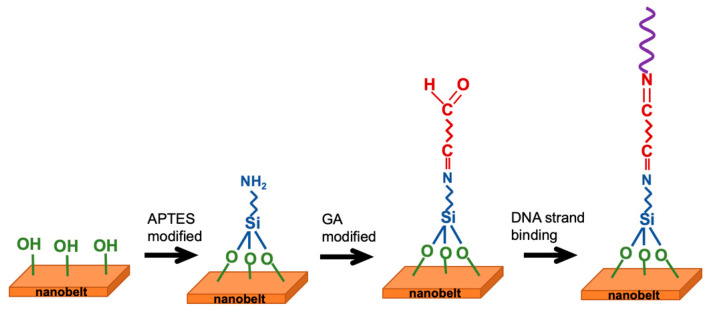
Schematic representation of surface functionalization and biografting steps.

The fabrication of the NBFET sensors involved a series of surface functionalizations to enable the attachment of biomolecules onto the nanobelt surfaces for biological sensing purposes. Prior to any chemical surface modification, the photoresist layer on the chips was removed by immersing them in a photoresist remover (EKC 830) at 90 °C for 15 min. Following this step, the chips underwent thorough cleansing and were subsequently dried. To introduce OH¯ terminals on the nanowire surfaces, an oxygen plasma treatment was administered for 5 min. Subsequently, the samples were immersed in a 10% aqueous solution of APTES for 30 min at 37 °C, with the pH adjusted to 3.5 using 1 M HCl. After this, the samples were rinsed with deionized (DI) water and dried on a hot plate at 120 °C for 30 min. The surface reactions primarily involved the silanol groups present on the silicon nanobelt surface due to silicon dioxide formation. Notably, silanol groups exhibit excellent proton donor (H^+^) and acceptor (SiO^–^) properties, making them suitable for binding with APTES to form a self-assembled monolayer on the nanobelt surface, thereby completing the NH_2_ surface functionalization [24]. Following the removal of excess alcohol, the amino groups were functionalized. To introduce aldehyde groups to the surface, GA was linked to the amino groups. The sample was immersed in a linker solution containing 2.5% GA for 30 min at room temperature and then rinsed with PBS solution. Figure 2 presents a scheme of the surface functionalization and biografting processes.

To bind the synthetic 15 mer single-stranded DNA to the nanobelt, a sequence dilution was conducted to provide a 10 nM solution of DNA using the PBS. One drop of the DNA solution was then placed on the nanobelt, then waiting 1 h to ensure complete binding, followed by a wash process to remove the unreacted DNA strands.

### 2.4. Fabrication of Microfluidic Channel

Before subjecting the NBFET biosensor to a long-term recording of its electrical response in a liquid environment, it was necessary to prepare the experimental setup. To facilitate this, a microfluidic channel was fabricated, featuring a width of 50 μm. This channel was constructed using polydimethylsiloxane (PDMS) and was securely sealed to the sensor device using a special fixture tool. The microfluidic channel served as a conduit for the flow of fluids through the nanobelt, allowing precise volume control and manipulation of the sensing environment. This controlled flow of target liquids was a critical aspect of the experiment setup and was achieved using an automated syringe pump, specifically Model 780270 (KD Scientific Inc., Holliston, MA, USA). This level of precision is crucial for long-time monitoring, particularly when studying the behavior of a biosensor’s stability and degradation in a liquid environment.

### 2.5. Device Measurement and Analysis

The fabricated nanobelt and NBFET surface morphology were observed by using a tunneling electron microscope (TEM) and a field-emission scanning electron microscope (FESEM), respectively. The electrical properties of the NBFET biosensor devices were characterized using an Agilent-4156C semiconductor parameter analyzer (Agilent Technologies, Santa Clara, CA, USA). The drain current versus gate voltage (*I_D_*–*V_G_*) curves of the NBFET devices were recorded in each step to examine their performance. Secondary ion mass spectroscopy (SIMS) was used to analyze ion penetration into the silicon. Surface roughness was inspected using an atomic force microscope (AFM).

## 3. Results and Discussions

**Figure 3:**
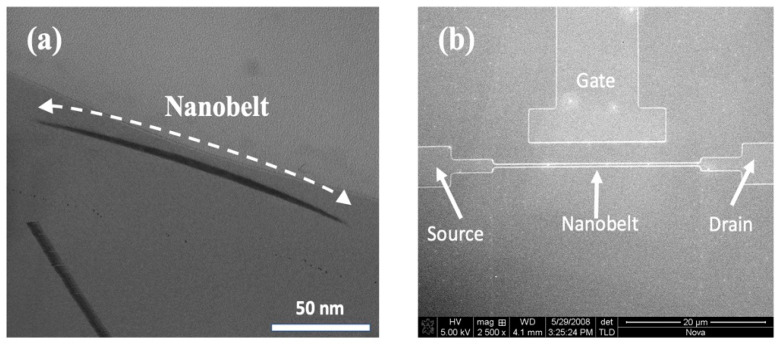
(**a**) Cross-sectional TEM images of the formed silicon nanobelt. (**b**) Top-view FESEM image of the NBFET structure.

We used a TEM (JEM-2010F, JOEL Ltd., Tokyo, Japan) to observe the structure of the nanobelt. Figure 3a presents a cross-sectional image of the silicon nanobelt, revealing key structural details. Notably, the width of the nanobelt underwent a significant reduction, diminishing from its original dimensions of 350 nm to a slender 150 nm. Moreover, the initial thickness of 50 nm experienced a substantial decrease to approximately 5 nm following the LOCOS processing, as visually demonstrated in the figure. It is imperative to underscore that the local oxidation of the silicon process, as shown in Figure 1d, is of critical importance in the formation of the nanobelt. This transformation in the nanobelt’s dimensions is intricately linked to the oxide thickness in the local oxidation process. Not only does the oxidation process completely oxidize the underlying silicon, but it also ensures the preservation of the nanobelt’s original thickness. This meticulous control over the structural changes in the nanobelt plays a pivotal role in enhancing the sensitivity of the NBFET biosensor. The improved sensitivity of the NBFET sensor is attributed to the substantial reduction in its dimensions while retaining a substantial detection region on the surface. This combination of factors contributes to the sensor’s heightened capability to detect and respond to subtle changes in its surroundings.

The morphology of the NBFET device was demonstrated through FESEM (JSM-6700F, JOEL Ltd., Tokyo, Japan). Figure 3b presents a top-view image of the NBFET device, including the source, drain, side gate, and nanobelt. The length of the nanobelt in our design is 30 μm long. The side gate was designed in the NBFET biosensor to provide an individual gate voltage for each device and hence ensure its controllable and stable properties.

**Figure 4:**
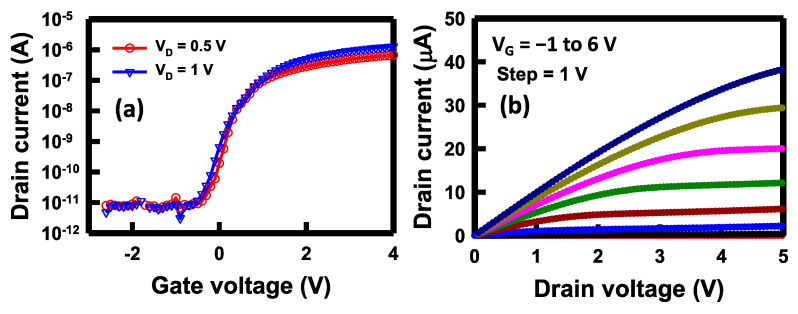
Fundamental electrical characteristics of the NBFET device. (**a**) *I*_D_–*V*_G_ curves at *V_D_* = 0.5 and 1 V, and (**b**) *I_D_*–*V_D_* curves at *V_G_* from −1 to 6 V.

To examine the electrical characteristics of the NBFET devices’ side gates, the drain current versus gate voltage (*I_D_*–*V_G_*) as well as the drain current versus drain voltage (*I_D_*–*V_D_*) were measured before the biosensing applications were conducted. The *I_D_*–*V_G_* and *I_D_*–*V_D_* curves serve as crucial indices for assessing the characteristics of a transistor device. Prior to electrical measurements, PBS was introduced through the microfluidic channel and subsequently remained on the nanobelt surface. It is noteworthy that the detection area was selectively opened on a segment of the nanobelt, as depicted in Figure 1g. The remaining portion of the entire device was shielded by a Si_3_N_4_/oxide film. In this configuration, only the nanobelt came into contact with the PBS, ensuring that the gate, drain, and source electrodes remained unaffected by the solution. The fundamental electrical performance of the transistor device is of paramount importance, as it directly impacts the biosensor’s sensitivity, limit of detection (LOD), as well as its stability and reliability when employed as a biosensor. In Figure 4a, we present the *I_D_*–*V_G_* plot at *V_D_* of 0.5 and 1 V. This plot illustrates the ability to switch the drain current (*I_D_*) flowing through the nanobelt, from 10^−11^ to 10^−6^ A, at various side-gate potentials. Under a substantial negative gate voltage, such as −2 V, the channel conduction is notably low. When applying a positive voltage to the gate, such as 2 V, it creates an electron channel, rendering the transistor normally on; thus, the current increases. In practice, the side gate can apply an electric field to the nanobelt, thus influencing the energy band for the charge carriers. The sensor’s practicality is further highlighted by the on/off current ratio, which approaches five orders of magnitude when *V_D_* is applied at 1 V. The determined threshold voltage (*V_t_*) can be extracted to approximately 0.5 V. The *V_t_* is defined as the minimum gate voltage required to create a conducting channel for electrons flowing from the source to drain, which can be extracted from the *I_D_*–*V_G_* curve. The subthreshold swing (*SS*) was calculated to be 150 mV/decade. This substantial ratio suggests the enhanced utility of the fabricated NBFET biosensors, which can be attributed to the heightened sensitivity.

Figure 4b depicts the *I_D_*–*V_D_* curves of the NBFET device across a range of applied V_G_ values, spanning from −1 to 6 V. It is observed that the *I_D_* increases when the *V_G_* increases, whereas only a modest increase occurs with increasing *V_D_*_._ The result in this figure highlights the distinctive behavior of *I_D_* concerning *V_D_* and *V_G_* for the NBFET device, which indicates that the primary factor influencing the current is the variation in *V_G_*. Therefore, the judicious selection of an appropriate gate voltage becomes imperative to operate the device under the most favorable conditions for subsequent biosensing applications. Understanding the interplay between *V_G_*, *V_D_*, and *I_D_* in this NBFET device is pivotal for optimizing its performance as a biosensor. By strategically adjusting the gate voltage, it becomes feasible to precisely modulate the device’s current response, enhancing its sensitivity and reliability in biosensing applications.

The stability of the NBFET device, especially when utilized as a biosensor in complex liquid environments, is a critical consideration. The challenges posed by extended exposure to buffer solutions, along with the presence of ions, necessitate a thorough investigation into the stability of the biosensor device. This endeavor is fundamental to ensuring the device’s reliability and effectiveness in real-world biomedical applications, where precise and consistent results are of utmost importance.

**Figure 5:**
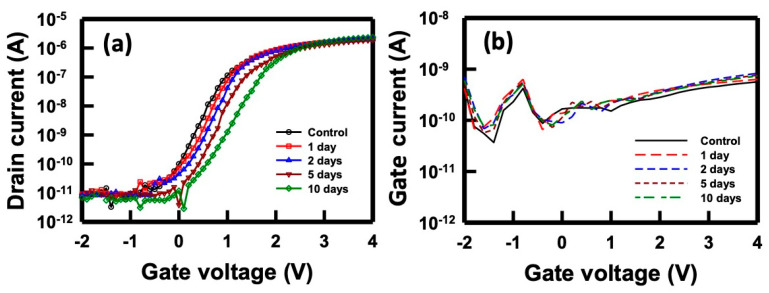
(**a**) *I*_D_–*V*_G_ and (**b**) *I*_G_–*V*_G_ curves of the NBFET biosensor immersed in PBS over time.

Given these intricate challenges, it was imperative to conduct a time-dependent stability assessment of the NBFET device when immersed in a buffer solution. This evaluation allowed us to gain insights into how the device’s performance evolves over time in real conditions. Illustrated in Figure 5a are the *I_D_*–*V_G_* curves of the NBFET biosensor immersed in PBS for varying durations. Evidently, the curves gradually shift right with increasing immersion time. Notably, the SS experienced a slight change after 5 days, becoming significantly smaller after 10 days of immersion. Figure 5b displays the *I*_G_–*V*_G_ curves of the devices immersed in the PBS. The gate leakage current remained consistently at the same level, indicating that the gate leakage was not the primary factor contributing to the device degradation over the 0- to 10-day immersion period. This observation underscores the impact of the buffer solution on the electrical characteristics of the NBFET device, revealing a heightened degradation as the immersion duration extends. These findings emphasize the necessity of understanding and addressing the time-dependent stability challenges in biosensor applications.

**Figure 6:**
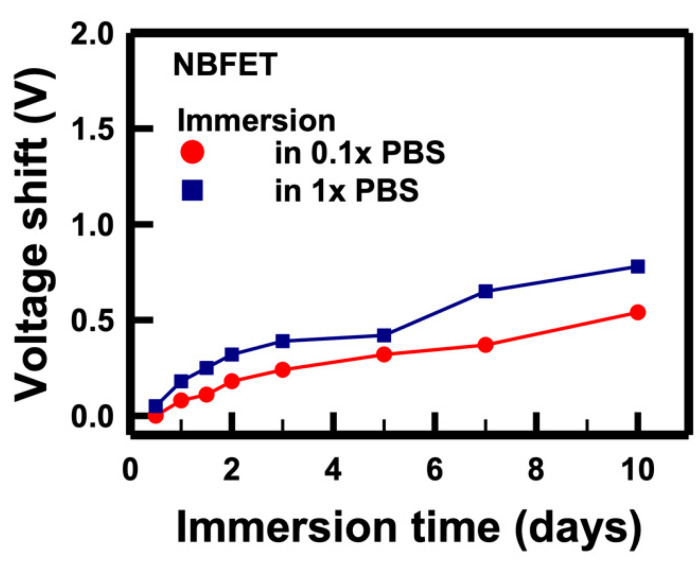
Voltage shifts of the NBFET biosensors immersed in PBS solutions with different concentrations over time.

To comprehend the mechanism underlying the degradation induced by the buffer solution, we conducted a thorough analysis of the electrical characteristics of the NBFET biosensors immersed in PBS solutions with varying concentrations. Figure 6 illustrates the voltage shifts of the NBFET devices immersed in a 10 mM (denoted as 1×) solution that was subsequently diluted to 1 mM (denoted as 0.1×). To calculate the voltage shift, we firstly extracted the *V_t_* value of the control sample, and then the *I_D_* was determined from the *I_D_–V_G_* curve. The *I_D_* served as the reference value (*I_D,ref_*), and the voltage shift was defined as the *V_G_* difference between two curves at *I_D,ref_*. The formula for this calculation is as follows:

(1) $$Voltageshift=(V_{G2}-V_{G,Control})\left|I_{D,ref}\right.$$

The results showed that, regardless of whether the devices were immersed in 1× or 0.1× solutions, the voltage shifts increased with prolonged immersion, aligning with the trends observed in Figure 5. Remarkably, a higher magnitude of voltage shifts was observed in the 1× PBS sample compared to that in the 0.1× PBS. This discrepancy can be attributed to the tenfold higher ion concentration in the 1× PBS compared to that in the 0.1× PBS. From this, we infer that the primary cause of NBFET degradation is the penetration of ions into the nanobelt structure during the immersion period. This understanding sheds light on the critical role of ion diffusion in the observed degradation of NBFET devices immersed in buffer solutions, further emphasizing the importance of addressing this challenge for enhanced device reliability and longevity.

**Figure 7:**
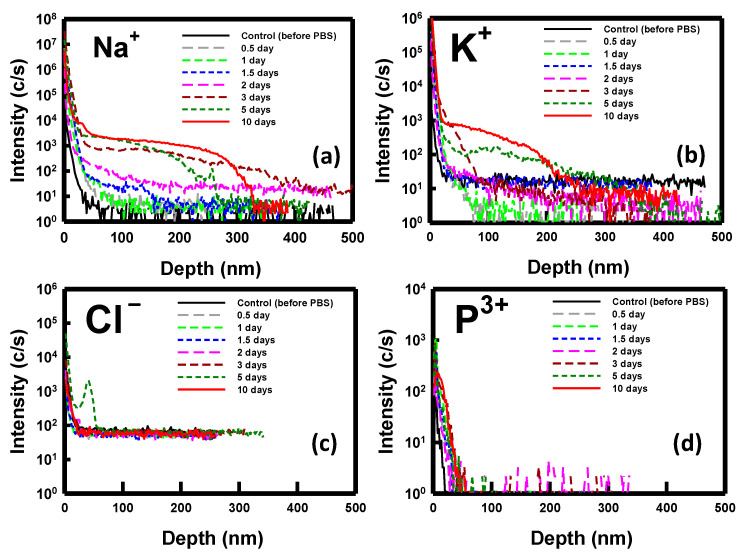
SIMS depth profile of the ions for the sample immersed in the PBS solution over various durations: (**a**) sodium ions, (**b**) potassium ions, (**c**) chlorine ions, and (**d**) phosphorus ions.

To elucidate the phenomenon of ion penetration during the immersion period, we employed SIMS (CAMECA IMS 7F, AMETEK, Inc., Gennevilliers, France) analysis for monitoring purposes. Analyzing the key ions present in the PBS solution, namely, sodium (Na^+^), potassium (K^+^), chlorine (Cl^−^), and phosphorous (P^3+^), we conducted a thorough investigation. Figure 7 depicts the SIMS depth profiles of these ions for samples immersed in PBS for durations ranging from 0.5 to 10 days. Notably, the measurements were performed on nondoped, bare silicon substrates. The results obtained highlight the penetration of Na^+^ and K^+^ ions into the substrate post-immersion, with no observable penetration of Cl^−^ and P^3+^ ions. Specifically, Na^+^ ions exhibited slight diffusion into the substrate after 2 days, intensifying from days 3 to 10. In contrast, a noticeable diffusion of K^+^ ions was observed after 5 days of immersion. These findings from the SIMS analysis align with the previously discussed electrical properties of the NBFET devices.

**Figure 8:**
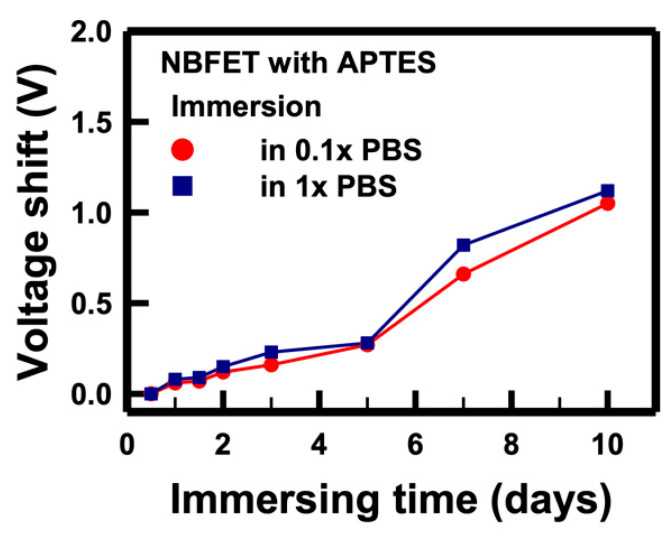
Voltage shifts of the APTES-functionalized NBFET biosensors immersed in PBS solutions with different concentrations over time.

The electrical performance of the NBFET biosensors featuring APTES coating on the nanobelt surface was also assessed to evaluate their effectiveness following PBS immersion. In Figure 8, the voltage shifts of the APTES-functionalized NBFET biosensors immersed in 1× and 0.1× PBS are illustrated. Notably, for samples subjected to less than 5 days of immersion, the APTES-functionalized NBFET devices exhibited smaller voltage shifts than their uncoated counterparts. However, the voltage shifts of the APTES-functionalized NBFET devices showed a significant increase during immersion from 7 to 10 days. Furthermore, the discrepancy in voltage shifts between 1× and 0.1× PBS conditions was found to be smaller for the APTES-functionalized samples compared to the uncoated ones. This observation leads us to conclude that, within the initial 5 days of immersion, ion penetration may influence the performance of NBFET devices. Beyond this period, however, ion penetration is not the predominant mechanism causing voltage shifts in the APTES-coated samples. These findings underscore the temporal dynamics of APTES-coating effectiveness in mitigating ion-induced effects on NBFET device performance.

**Figure 9:**
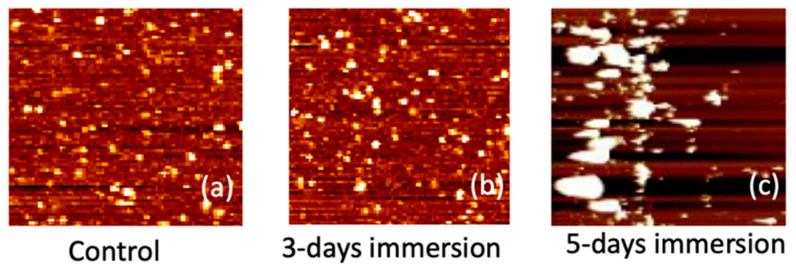
Surface AFM images of the APTES-coated samples for (**a**) control (0 days), (**b**) 3 days, and (**c**) 5 days of PBS immersion.

In Figure 9, the AFM images depicting the APTES-coated samples are presented for intervals of 0, 3, and 5 days of PBS immersion. The presence of white spots in the images signifies chemical molecules binding to the surface. Following a 3-day immersion, the number of white spots noticeably increased. Notably, after 5 days of immersion, the size of these white spots substantially increased, forming clusters on the sample surface. We hypothesize that these white clusters resulted from APTES peeling off after prolonged immersion in the PBS solution. This supposition aligns with the electrical measurement results showcased in Figure 8. Given the positively charged nature of the APTES film, the peeling off of the film induces a rightward shift in the *I_D_*–*V_G_* curve of the NBFET device, leading to an increase in *V_t_*. This correlation between the AFM observations and the electrical measurements provides valuable insights into the impact of prolonged PBS immersion on the APTES-coated samples, further underlining the intricate dynamics between surface modifications and device performance.

**Figure 10:**
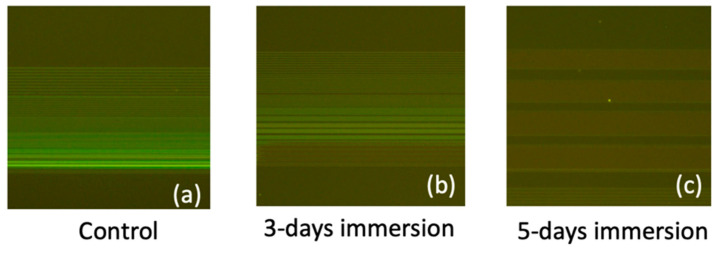
Fluorescence images of the FITC-labeled DNA strands bonded to the SiO_2_ line patterns for PBS immersion durations of (**a**) control (0 days), (**b**) 3 days, and (**c**) 5 days.

In Figure 10, fluorescence images depicting FITC-labeled DNA strands bonded to the SiO_2_ line patterns are presented for immersion durations of 0, 3, and 5 days in PBS. The attachment of DNA strands to the SiO_2_ line patterns was facilitated through amine and carboxyl interactions, achieved via the APTES and GA functionalization process. Notably, the control sample exhibited the highest and most distinct fluorescence intensity, indicating a high binding efficiency of the DNA strands to the SiO_2_ substrate. However, the fluorescence intensity decreased as the immersion duration increases. By integrating the results from the AFM and fluorescence images, we deduced that prolonged PBS immersion induces the peeling off of the APTES film, consequently degrading the binding efficiency of biomolecules. This comprehensive analysis underscores the intricate interplay between surface modifications, biomolecule binding, and the environmental factors affecting biosensor performance over time.

## 4. Conclusions

This paper presents an exploration of the electrical properties of NBFET biosensor devices exposed to buffer solutions for extended durations. Fabricated through a CMOS-compatible manufacturing process, the NBFET biosensor features a nanobelt formed using the local oxidation of silicon. The dimensions of the nanobelt are 200 nm wide and 30 μm long, with a thickness of 10 nm. Employing this technique, the NBFET device exhibits a remarkable five orders of magnitude increase in on/off current ratio and a subthreshold swing of 150 mV/decade.

Upon immersion in a buffer solution, the results indicate that the buffer solution accelerates the degradation of the transistor device, leading to an increase in the threshold voltage shift with prolonged immersion. SIMS analysis further confirms the penetration of Na^+^ and K^+^ ions into the substrate after 5 days of PBS immersion. Additionally, for the APTES-coated NBFET biosensor devices, PBS immersion is shown to cause the deterioration of surface functionalization.

In summary, the infiltration of the buffer solution into the device raises concerns regarding the reliability of the biosensor and the potential deterioration of surface functionalization after prolonged immersion. This study sheds light on the dynamic behavior of NBFET devices in real conditions, emphasizing the importance of addressing these challenges for enhanced biosensor performance and longevity.

## Data Availability

Data are contained within the article.

## List of Acronyms

APTES	3-amino-propyl-triethoxy-silane
AFM	atomic force microscope
C_2_H_5_OH	ethanol
DI	deionized
FET	field-effect transistor
FITC	fluorescein isothiocyanate
FESEM	field-emission scanning electron microscope
GA	glutaraldehyde
*I_D_*–*V_G_*	drain current versus gate voltage
*I_D_*–*V_D_*	drain current versus drain voltage
LOD	limit of detection
NBFET	nanobelt field-effect transistor
PBS	phosphate-buffered saline
PDMS	polydimethylsiloxane
SOI	silicon-on-insulator
SiO_2_	silicon oxide
Si_3_N_4_	silicon nitride
SIMS	secondary ion mass spectroscopy
*SS*	subthreshold swing
TSRI	Taiwan Semiconductor Research Institute
TEM	tunneling electron microscope
*V_t_*	threshold voltage
